# Decellularized cryopreserved human pericardium: a validation study towards tissue bank practice

**DOI:** 10.1007/s10561-023-10072-6

**Published:** 2023-01-25

**Authors:** Giulia Montagner, Antonia Barbazza, Andrea Tancredi Lugas, Mara Terzini, Gianpaolo Serino, Cristina Bignardi, Matilde Cacciatore, Vladimiro L. Vida, Massimo A. Padalino, Diletta Trojan

**Affiliations:** 1 Fondazione Banca Dei Tessuti del Veneto Onlus, Treviso, Italy; 2 PolitoBIOMed Lab, Politecnico Di Torino, Turin, Italy; 3 Department of Mechanical and Aerospace Engineering, Politecnico Di Torino, Turin, Italy; 4 Unità Operativa Complessa Anatomia Patologica, AULSS2 Marca Trevigiana, Ospedale Di Treviso, Treviso, Italy; 5 Pediatric and Congenital Cardiac Surgery Unit, Department of Cardiac, Thoracic and Vascular Sciences and Public Health, University of Padua, Padua, Italy

**Keywords:** Pericardium, Allograft, Decellularization, Tissue bank, Cardiac septal defects

## Abstract

Pericardial patches are currently used as reconstructive material in cardiac surgery for surgical treatment of cardiac septal defects. Autologous pericardial patches, either treated with glutaraldehyde or not, can be used as an alternative to synthetic materials or xenograft in congenital septal defects repair. The availability of an allogenic decellularized pericardium could reduce complication during and after surgery and could be a valid alternative. Decellularization of allogenic tissues aims at reducing the immunogenic reaction that might trigger inflammation and tissue calcification over time. The ideal graft for congenital heart disease repair should be biocompatible, mechanically resistant, non-immunogenic, and should have the ability to growth with the patients. The aim of the present study is the evaluation of the efficacy of a new decellularization protocol of homologous pericardium, even after cryopreservation. The technique has proven to be suitable as a tissue bank procedure and highly successful in the removal of cells and nucleic acids content, but also in the preservation of collagen and biomechanical properties of the human pericardium.

## Introduction

The pericardium is a connective sac with parietal and visceral component, which has a protective and mechanical role. In fact, it maintains the geometry of the heart and its pressure–volume correlation. In particular, the parietal pericardium is made up of mesothelial cell and a fibrous layer, that is composed mainly by collagen fibres (Rodriguez and Tan 2017).

Porcine and bovine pericardial patches have been used for decades as xenograft reconstructive material in cardiac surgery for heart valve repair, atrial or ventricular septal reconstruction, as well as for vessel reconstruction in vascular surgery. Also, it has been used in non-cardiac surgical procedures such as abdominal wall repair and dural repair (Rémi et al. 2011).

Usually, these xenografts are crosslinked, mainly with glutaraldehyde (GA); however, the fixation is associated with cytotoxicity and tissue calcification after transplantation (Grabenwoger et al. 1996; Ma et al. 2014). For these reasons, different chemical crosslinking methods have been proposed (Jorge-Herrero et al. 2010).

An alternative preparation of pericardium xenografts is the decellularization, that aims at removing antigenicity and immune response after transplantation, while preserving the extracellular matrix (ECM) integrity and the mechanical resistance.

Allogenic ECM is the ideal graft to minimize immunogenicity after implantation in recipients. Nevertheless, decellularization of allogenic tissues aims at reducing the immunogenic reaction that might trigger inflammation and tissue calcification over time, as reported for allogenic cardiac valves (Carr-White et al. 2001, Hogan et al. 2003).

Decellularization protocols are usually based on chemical or physical treatments or a combination of both. Chemical procedures include incubation in enzymes, ionic agents or detergents, while physical treatments are commonly agitation, freeze-thawing and sonication (Gilbert et al. 2006).

Recently, several decellularization protocols of human pericardium have been reported with the aim to avoid immunogenic reactions in the recipient. The decellularization protocol with sodium dodecyl sulphate and Ethylenediaminetetraacetic acid reported by Wollmann et al. achieved the removal of cells without affecting the biomechanical properties of the tissue and avoiding cytotoxicity (Wollmann et al. 2019). However, the residual DNA reported is tenfold higher than the limit of 50 ng/mg of dry tissue recommended by Crapo et al. (Crapo et al 2011). The decellularization method developed by Mirsadree et al. is based on consecutive incubations in hypotonic buffer, sodium dodecyl sulphate (SDS) in hypotonic buffer and nucleases (RNase/DNase) (Mirsadraee et al. 2006). Advantages of this decellularized pericardium have been demonstrated in vivo in comparison with both fresh/frozen and glutaraldehyde-fixed pericardium, with improvement in integration, regeneration and lack of calcification (Mirsadraee et al. 2007). However, clinical data about the use of this pericardial patches in patients are still lacking.

The aim of the present study is the evaluation of the efficacy of a new decellularization protocol, in particular to assess the effective removal of cells and nucleic acids content of human pericardia, while maintaining its biomechanical properties and collagen extracellular content, even after cryopreservation.

## Methods

### Tissue procurement

Human pericardia were procured from cadaver donor following Italian directives and with the proper informed consent. Only pericardia unsuitable for transplantation but morphologically unaltered were utilized for the protocol set up and validation. The retrieval was performed within 24 h of cardiac arrest or 12 h if the cadaver was not refrigerated during the first six hours after death. After retrieval, pericardia were transferred in BASE medium (Alchimia srl, Italy) containing gentamicin 200 µg/ml (Fisiopharma, Italy), vancomycin 100 µg/ml (Pharmatex, Italy) and meropenem 200 µg/ml (Fresenius Kabi AG, Germany), a solution validated for tissues decontamination (Serafini et al. 2016, Paolin et al. 2018, Montagner et al. 2018). Tissues were subsequently transported to the tissue establishment at + 4 °C.

### Tissue process and cryopreservation before decellularization

After of the first decontamination, pericardia were processed in cleanrooms; all the following steps were carried out in grade A cabinet flow hood in grade B laboratory. Adipose residues were removed from the pericardia that were subsequently transferred in BASE medium (Alchimia srl, Italy) containing antibiotics for minimum 48 h, according to the standard operating procedure for pericardia applied in our tissue bank. In order to facilitate tissue management and to optimize the utilization rate of pericardial patches, we decided to cryopreserve the patches before decellularization. At the end of the second decontamination, pericardia were packaged in sterile ethylene vinyl acetate bags (Agricons Ricerche, Italy) and immersed in a solution containing BASE medium (Alchimia srl, Italy), 10% DMSO (WAK-Chemie Medical GmbH, Germany) and 2% human albumin (Behring, Italy). Before cryopreservation in vapor phase liquid nitrogen, a programmable cryogenic freezer Planer KryoSave Integra 750–30 (Planer Limited, UK) was used for the controlled cooling rate.

### Thawing before decellularization

Before decellularization, pericardia were defrosted inside the sterile double bag in warm water (37 °C), until completely thawed. Tissues were then washed in sterile saline solution.

### Decellularization

Pericardia decellularization were carried out in three consecutive days. The protocol is patent pending and is based on a hypertonic solution and two reagents, benzonase (Sigma-Aldrich, USA) and sodium cholate (Sigma-Aldrich), which were already utilized in our tissue bank and approved by the national competent authority for the decellularization of dermis. At the end of the decellularization, pericardia were transferred in BASE medium (Alchimia srl, Italy) containing gentamicin 200 µg/ml (Fisiopharma, Italy), vancomycin 100 µg/ml (Pharmatex, Milan, Italy) and meropenem 200 µg/ml (Fresenius Kabi AG, Germany) overnight. The following day, pericardia were cryopreserved as previously described.

### Microbiological analysis

Several microbiological tests were conducted throughout the pericardia process in order to verify the compliance with the acceptance criteria and regulation (Table 1). Samples were inoculated and incubated in BD BACTEC culture vials, in accordance with the manufacturer’s instructions (BD, Becton, Dickinson and Company, USA). If the samples tested positive, the microorganisms were isolated and identified using standard procedures. Moreover, environmental monitoring was conducted according to national directives.

**Table 1:** Acceptance criteria for the tissue bank process

	Step of microbiological analysis	Acceptance criteria
1	Process before decellularization	Negative culture or absence of microorganisms that results in tissue discard according to a risk analysis
2	Cryopreservation before decellularization	Negative culture
3	End of decellularization	Negative culture or absence of microorganisms that results in tissue discard according to a risk analysis
4	Cryopreservation after decellularization	Negative culture

### Histological analysis and fluorescence staining of nuclei

Samples of decellularized and native pericardia were embedded in Optimal cutting temperature compound (OCT compound, Kaltek, Italy) using PrestoCHILL (Milestone Medical, Italy). Tissue blocks were then sliced into 6 µm sections, which were fixed in 95% ethanol, rehydrated with 70% ethanol and stained with haematoxylin and eosin (Merck, Germany). For nuclei fluorescence staining, tissue sections were fixed in ethanol 95% and stained with Hoechst 33,342 (Thermo Fisher Scientific, USA). Pictures were taken using the light and fluorescent Leica DMi8 microscope equipped with camera (Leica Microsystems, Germany).

### DNA residual analysis

After thawing and freeze-drying, 10 mg of each sample was weighed for DNA extraction, which was achieved using QIAamp DNA Mini Kit (Qiagen, Germany), following manufacturer’s instructions. DNA concentrations were measured with Qubit 4 fluorometer (Thermo Fisher Scientific, USA), using the Qubit™ 1X dsDNA High Sensitivity (Thermo Fisher Scientific, USA). Moreover, the presence of DNA fragments was observed by gel electrophoresis, using the GeneRuler Express DNA Ladder (Thermo Fisher Scientific, USA) as a marker.

### Collagen quantitative analysis

After thawing, each sample was weighed and digested with 0.1 mg/ml pepsin (Sigma-Aldrich, USA) in 0.5 M acetic acid (Merck, Germany). Collagen quantification was performed by Sircol S1000 assay (Biocolor, UK) following manufacturer’s instruction. Quantification was performed using the Byonoy absorbance microplate reader (Byonoy GmbH, Germany).

### Mechanical tests

Three decellularized patches and three non-decellularized patches, obtained from three different donors were mechanically tested. Before the mechanical test, each patch was immersed (including packaging) in water at 37 °C until the complete melting of the conservation medium.

**Fig. 1:**
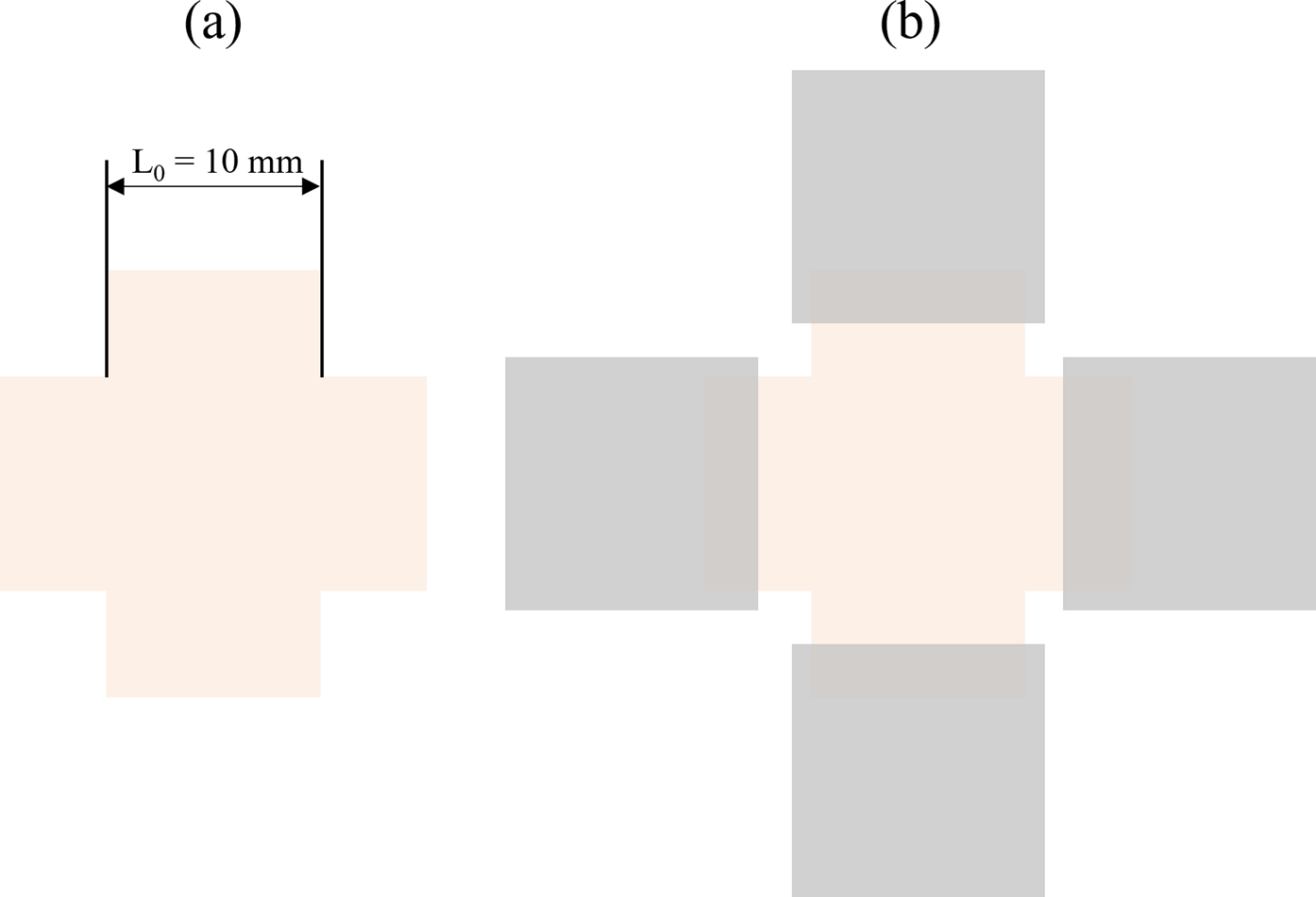
Cruciform specimen representation (**a**) and gripping (**b**)

Biaxial mechanical tests were conducted in order to simultaneously explore the decellularization treatment effect and the pericardium anisotropy. The cruciform specimens (Fig. 1a) were obtained from each patch using a custom cutting tool.

The specimen thickness was determined as the mean of three measurement taken with a thickness gage (547–321, Mitutoyo, Lainate, Italy) in three points in the central region of each specimen.

An equi-biaxial test, characterized by the same stretch applied in two orthogonal directions, was performed using a biaxial tensile machine (Planar Biaxial, TA Instruments, New Castle, USA) in displacement control, with a constant elongation rate of 0.32 mm/s while measuring the resulting force with a 225 N load cell. The specimen arms were inserted and gripped in the pressure jaws of the tensile test machines, leaving the central region unconstrained (Fig. 1b), and an elongation of 2 mm was imposed along each direction starting from the non-deformed condition of the specimens.

From each test, a stress–strain curve was obtained and used to compute the elastic modulus of each specimen (as the slope of the stress–strain curve in its linear region). Namely, stress is the mean value of the load exerted on the section perpendicular to the load direction, and strain is the elongation relative to the initial length. The two quantities were calculated as follow:

$$\sigma =\frac{F}{A} ; \varepsilon =\frac{L-{L}_{0}}{{L}_{0}}$$

where $A$ is the mean cross section, calculated as $A=t{L}_{0}$, ${L}_{0}$ is the initial length of the two sides of the specimen and, $L$ is the length of the stretched sides, and $t$ is the specimen thickness. The elastic modulus was determined as the slope of the stress–strain curve in its linear region, according to the following equation:

$$\sigma =E\varepsilon $$

Being the direction of the extracellular matrix fibers unknown, the loading directions presenting the lowest and highest elastic moduli of each specimen will be referred as to Direction 1 and Direction 2 in the following.

We also evaluated whether mechanical properties were different between the three batches of pericardium analysed, retrieved from three different donors.

### Statistical analysis

In DNA and collagen quantification the t single sample test was used and *p* values less than 0.05 (*p* < 0.05) were considered statistically significant. In other to investigate the influence of the donor and the treatment on the human pericardium elastic modulus, one-way ANOVA and pairwise t-test were used, respectively.

## Results

### Microbiological analysis

All the microbiological tests were compliant with the acceptance criteria. All the environmental controls were negative, demonstrating that the entire process is achievable in a Good Manufacturing Practice (GMP) compliant facility and as a tissue bank practice.

### Histology and fluorescence nuclear staining

**Fig. 2:**
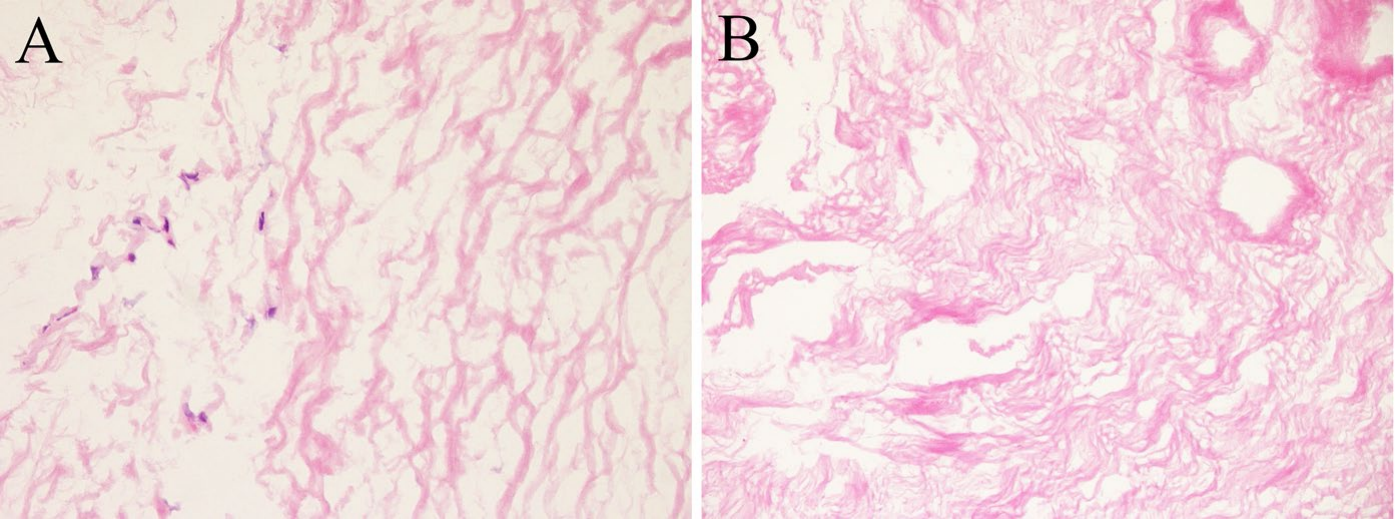
Haematoxylin and eosin staining of representative histological sections of non-decellularized pericardium (**a**) and decellularized pericardium (**b**), 40 × magnification Absence of nuclei is demonstrated in decellularized samples. Extracellular matrix is similar between non-decellularized pericardium (**a**) and decellularized pericardium (**b**)

**Fig. 3:**
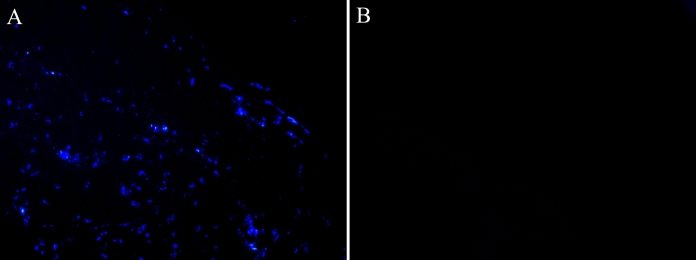
Nuclear fluorescence staining with Hoechst in representative samples of non-decellularized pericardium (**a**) and decellularized pericardium (**b**). Nuclei are absent in decellularized sample. 20 × magnification

Haematoxylin and eosin staining of pericardia samples showed the maintenance of the extracellular matrix in decellularized samples (DPCs) compare to non-decellularized ones (NDPCs) (Fig. 2). Nuclei were absent in decellularized sample, while they were identifiable in native samples, as demonstrated both by haematoxylin and eosin staining and Hoechst staining (Fig. 3).

### DNA residual analysis

**Fig. 4:**
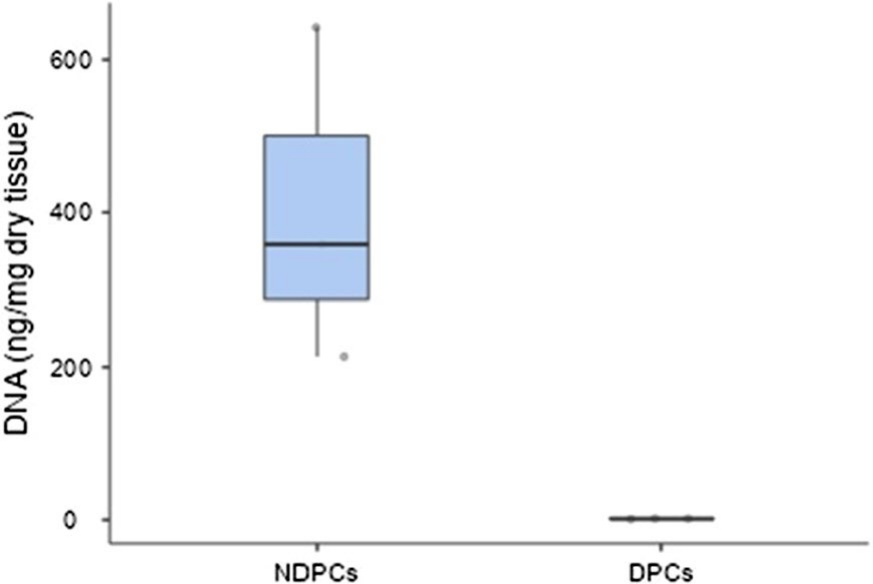
DNA quantity in non-decellularized samples (NDPCs) and decellularized ones (DPCs), obtained from three batches of pericardium (3 different donors). The reduction was statistically significant (p < 0.001) and the residual DNA in DPCs was below the limit of 50 ng/mg of dry tissue

**Fig. 5:**
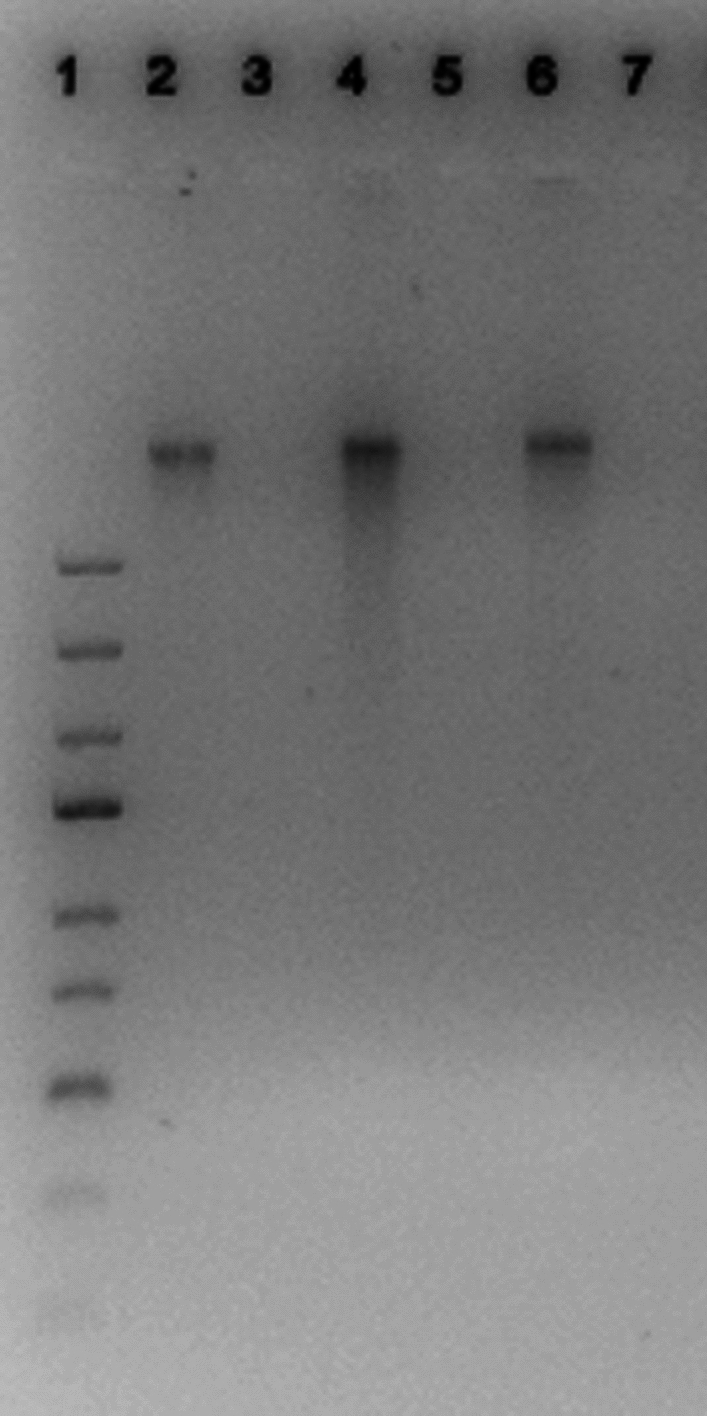
Electrophoresis analysis of nuclei acids compared to a marker (1). The image demonstrates the presence of DNA in non-decellularized samples (2, 4, 6) and the absence of nucleic acids fragments in decellularized pericardia samples (3, 5, 7). A total of three different batches (three different donors) of pericardia were included in the analysis

The quantification of residual DNA in DPCs detected about 2 ng/mg of dry tissue of DNA, ten-fold below the limit of 50 ng/mg of dry tissue required for an efficient decellularization (Crapo et al. 2011). Figure 4 reports the DNA quantity of DPCs and NDPCs samples of three batches of pericardium. Moreover, electrophoresis analysis demonstrated the absence of nucleic acids fragments in DPCs of the three batches of pericardium (Fig. 5).

### Collagen quantitative analysis

The quantification of collagen demonstrated that the decellularization did not alter the extracellular matrix; in the three different batches of pericardium analysed (three different donor), no statistical reduction of collagen content was observed in NDPCs compared to DPCs of the same batch (Table 2).

**Table 2:** Collagen quantity in non-decellularized samples (NDPCs) and decellularized ones (DPCs), obtained from three batches of pericardium (3 different donors). No statistical reduction of collagen content was observed in NDPCs compared to DPCs

	NDPCs	DPCs
Batch 1	1.52 ± 0.0721	2.09 ± 0.0699
Batch 2	1.24 ± 0.0068	1.55 ± 0.0623
Batch 3	1.31 ± 0.0225	2.58 ± 0.3510

### Mechanical test

**Fig. 6:**
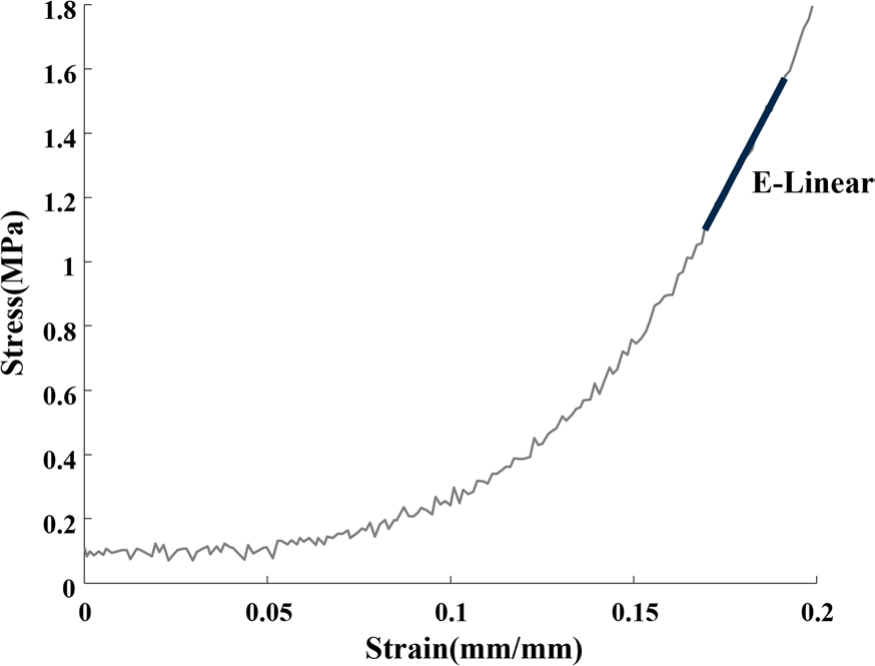
Representative experimental stress–strain curve and identification of linear region for elastic modulus estimation

An explanatory stress–strain curve is reported in Fig. 6, indicating the linear portion of the curve used to calculate the elastic modulus for both the directions.

**Fig. 7:**
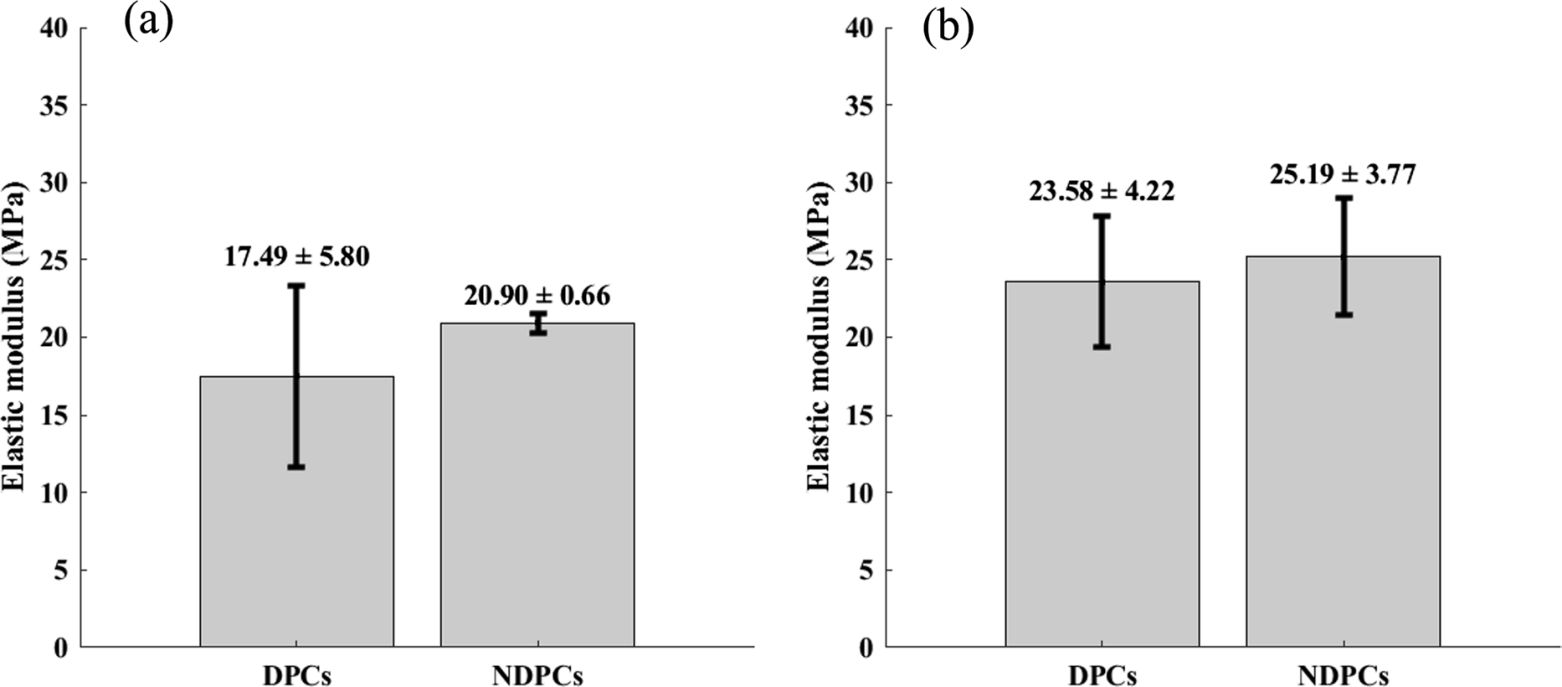
Treatment comparison along Direction 1 (**a**) and Direction 2 (**b**). The elastic modulus is not statistically different between decellularized samples (DPCs) and non-decellularized ones (NDPCs)

**Fig. 8:**
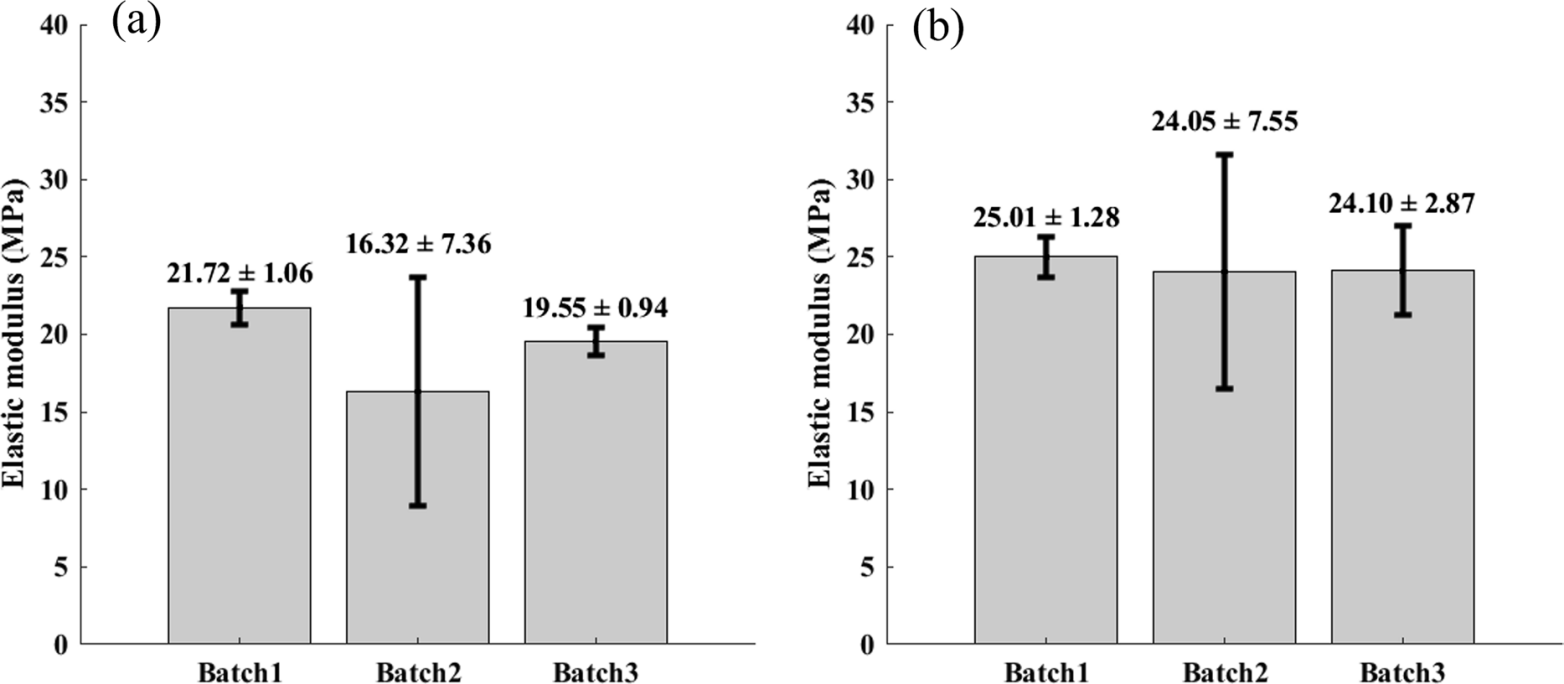
Donor comparison along Direction 1 (**a**) and Direction 2 (**b**). Non statistical differences in the elastic modulus were observed between the three different batches

The mean and standard deviation values of the elastic modulus are reported in Fig. 7 grouped according to the treatment, while Fig. 8 shows the donor comparison where the mean and standard deviation values of the elastic modulus are computed gathering results according to donors. Comparisons were performed differentiating the two directions, thus focusing the analysis on the material anisotropy.

No significant differences were highlighted between DPCs and NDPCs, neither along Direction 1 nor along Direction 2.

Similarly, the different donors did not significantly influence the elastic modulus of the pericardium along the two directions.

## Discussion

Autologous and homologous pericardial patches are currently the best options for surgical treatment of cardiac septal defects. The ideal graft for congenital heart disease repair should be biocompatible, mechanically resistant, non-immunogenic, and should have the ability to growth with the patients. Autologous pericardial patches, either treated with glutaraldehyde or not, can be used as an alternative to synthetic materials or xenograft in congenital septal defects repair. In recent years, several studies have reported clinical outcomes of septal repair with autologous pericardium patch. In 2020 IJsselhof et al. reported that ventricular septal defects can safely be closed with either untreated autologous pericardium or xeno-pericardium, with no difference in left atrio-ventricular valve regurgitation or need for reoperation; the study was conducted on 77 paediatric patients (median age 3.6 months) and the median follow-up was 17.5 years (IJsselhof et al. 2020). A retrospective study on 156 patients with a median follow-up of 37 months have demonstrated that autologous pericardium treated with glutaraldehyde is as effective as Dacron mesh in ventricular septal defects repair, with no differences in clinical outcomes and reinterventions (Desai et al. 2022).

The availability of an allogenic decellularized pericardium could reduce complication during and after surgery and could be a valid alternative if autologous pericardium can’t be used. The results of use of cryopreserved homologous pericardium treated with glutaraldehyde for surgical repair of cardiac diseases have been published recently. A total of 134 patients with a median age of 1.47 years have been recruited. Cryopreserved homologous pericardium was applied in aorta, interventricular septum, interatrial septum and pulmonary arteries. Glutaraldehyde fixation have been carried out with the aim to ease tissue handle, prevent aneurysmal dilation and to cross-link the collagen. However, in this experience, there is a high rate of grafts failures (19.4% of patients), which have been attributed to glutaraldehyde treatment, that causes tissue calcification (Gluck et al. 2022).

Cryopreservation of decellularized human pericardium has been suggested and studied by other authors, with the aim of render it easily available in tissue banks and clinical practice (Vinci et al. 2013); the decellularization method was similar to the protocol developed by Mirsadree et al. (Mirsadraee et al. 2006). However, no clinical data have been published so far.

We decided to investigate the efficacy of our decellularization method, already approved by our competent authority for the decellularization of human dermis. The technique has proven to be highly successful in the removal of cells and nucleic acids content, but also in the preservation of collagen and biomechanical properties of the human pericardium. Cytotoxicity of the reagents has been investigated previously and has not been detected (data not shown). The protocol has been established considering the feasibility in a tissue bank practice; for that reason, pericardial patches were cryopreserved before and after the decellularization. It is of note that, in spite of that, all the biomechanical properties and collagen content of these patches were unaltered. We believe that this finding is extremely important, since the procedure facilitates the management of pericardial patches in tissue bank practice and optimizes the utilization rate of them.

In the clinical cardiac surgery setting, especially in paediatric field, the durability of a graft is of paramount importance, since it determinates the risk of reoperation, so it can substantially modify (improving) the patient’s prognosis. For such a reason, we advocate the necessity of a continuous research in this field, so as to ameliorate the quality of replacing materials and possibly minimize the risk for reoperations. In this direction, the availability of the decellularized allogenic pericardium may represent a valid innovation for future clinical improvement. Further studies are needed to investigate the in vivo safety and effectiveness of this grafts.

## Data Availability

The datasets analysed during the current study were collected in Fondazione Banca dei Tessuti del Veneto and will be available on reasonable request.
